# Preoperative Burosumab With Delayed FGF23 Recovery and High Postoperative Bone Turnover in Tumor-Induced Osteomalacia

**DOI:** 10.1210/jcemcr/luaf296

**Published:** 2026-01-27

**Authors:** Heng Yeh, Htoo Myat Nge, Asmita Ghimire, Chelsea Gordner

**Affiliations:** Department of Medicine, Bassett Medical Center, Cooperstown, NY 13326, USA; Memorial Healthcare System, Internal Medicine Residency Program, Pembroke Pines, FL 33028, USA; Memorial Healthcare System, Internal Medicine Residency Program, Pembroke Pines, FL 33028, USA; Department of Adult and Pediatric Endocrinology, Memorial Healthcare System, Hollywood, FL 33021, USA

**Keywords:** tumor-induced osteomalacia, burosumab, fibroblast growth factor 23, high bone turnover, hungry bone syndrome

## Abstract

Tumor-induced osteomalacia (TIO) is a rare paraneoplastic syndrome caused by the overproduction of fibroblast growth factor 23 (FGF23) from phosphaturic mesenchymal tumors (PMTs). Clinical features include skeletal deformities, bone mineral density (BMD) loss, and debilitating myopathy. Hypophosphatemia and low 1,25(OH)_2_D levels are hallmark biochemical findings. We report a 46-year-old man with delayed tumor localization who received preoperative burosumab. Postoperatively, he developed transient mild hypocalcemia, persistently elevated alkaline phosphatase and parathyroid hormone, and prolonged FGF23 elevation for 6 months despite normalized serum phosphate and improved BMD. Burosumab can interfere with FGF23 assays and may cause extremely high in vivo FGF23 values for months. Alternative biochemical markers should be pursued postoperatively in patients who received burosumab before surgery. A high bone turnover state resembling hungry bone syndrome may occur after PMT resection. Awareness of these postoperative biochemical changes and the effects of burosumab on FGF23 assays is essential for monitoring TIO recovery.

## Introduction

Tumor-induced osteomalacia (TIO) is a rare paraneoplastic syndrome caused by the overproduction of fibroblast growth factor 23 (FGF23) from phosphaturic mesenchymal tumors (PMTs). The prevalence is 0.43 per 100 000 adults, with a slight male predominance (56.6%) and a mean age of 46.3 years [1, 2]. Excessive FGF23 downregulates sodium-phosphate cotransporters in the renal proximal tubule, leading to renal phosphate wasting and suppressing 1α-hydroxylase expression, resulting in persistent hypophosphatemia and low active 1,25-dihydroxyvitamin D (1,25(OH)_2_D) [1, 2].

The symptoms of TIO are often nonspecific and can lead to delayed or incorrect diagnosis, with an average time from symptom onset to diagnosis of 4.8 years [2]. Common manifestations include bone pain, fractures, and skeletal deformities such as height loss, kyphosis, and pectus carinatum, while nonskeletal manifestations include fatigue, generalized myalgia, and weakness [1].

The hallmark biochemical features of TIO are persistent hypophosphatemia (99.8%) and low 1,25(OH)_2_D (69.8%). Calculation of the tubular maximum phosphate reabsorption per glomerular filtration rate (TmP/GFR) and measurement of FGF23 help confirm renal phosphate wasting and establish FGF23-related disorder. Functional imaging studies, including ^68^Ga-DOTATATE positron emission tomography/computed tomography (PET/CT) and octreotide scans, are essential for localizing the culprit tumor for surgical resection [1].

Surgical removal of the tumor remains the definitive treatment. Serum FGF23 concentrations typically normalize within hours after complete tumor removal, followed by normalization of phosphate and 1,25(OH)_2_D within 5 days, and gradual improvement in bone mineral density (BMD) over 2 to 4 years. For patients who have unresectable, unlocalized, or recurrent tumors, conventional therapy with oral phosphate and calcitriol supplementation, as well as anti-FGF23 antibody therapy with burosumab, are alternative treatment options [1]. Previous reports have noted delayed normalization of FGF23 following tumor removal during ongoing burosumab therapy [3].

Following tumor resection, a period of high bone turnover with hypocalcemia, persistently elevated alkaline phosphatase (ALP), and worsening of secondary hyperparathyroidism has been described in a few reports [4-6]. These biochemical changes resemble hungry bone syndrome, a complication observed after parathyroidectomy in patients with severe hyperparathyroidism [7].

We present a patient case of TIO with delayed tumor localization who was treated preoperatively with burosumab and demonstrated prolonged postoperative elevation of FGF23 for up to 6 months, accompanied by high bone turnover, transient mild hypocalcemia, elevated ALP, and secondary hyperparathyroidism.

## Case Presentation

A 46-year-old man, with a history of cervical spinal stenosis and lumbar spondylosis, status post cervical spinal fusion 4 years prior, presented for evaluation of metabolic bone disease. He had bilateral hip pain, and imaging revealed bilateral subtrochanteric stress fractures and multiple rib fractures without significant trauma. The bone density testing revealed a T-score of −3.5 at the left femoral neck, −3.1 at the left total hip, and −2.6 at the lumbar spine (L1-L4). With low BMD and a history of fragility fractures, he was diagnosed with osteoporosis. He also reported generalized myalgia, weakness, fatigue, and unintentional weight loss of 100 pounds (∼45 kg) since the cervical spine surgery. He required a walker for ambulation and had no family history of metabolic bone disorder or osteoporosis. Physical examination revealed diffuse muscle weakness with normal deep tendon reflexes. He was unable to stand from a seated position without assistance.

## Diagnostic Assessment

The initial biochemical evaluation is shown in Table 1. Laboratory results showed new-onset hypophosphatemia 1.4 and 1.6 mg/dL (SI: 0.45 and 0.52 mmol/L) (reference range, 2.5-4.5 mg/dL [SI: 0.81-1.45 mmol/L]), low 1,25(OH)_2_D 16 pg/mL (SI: 38.4 pmol/L) (reference range 18-72 pg/mL [SI: 43-87 pmol/L]), elevated ALP, and parathyroid hormone (PTH) levels, with normal calcium and 25(OH)D. One year earlier, his serum phosphate was 2.8 mg/dL (SI: 0.90 mmol/L). His 24-hour urinary calcium and creatinine were low, excluding primary hyperparathyroidism. Further testing revealed a low TmP/GFR 1.24 mg/dL (SI: 0.4 mmol/L) (reference range, 2.6-4.4 mg/dL [SI: 0.8-1.35 mmol/L]) and fractional tubular phosphate reabsorption of 77% (reference range >85% in hypophosphatemia), consistent with urinary phosphate wasting. Plasma FGF23 was elevated at 356 RU/mL (reference range <180 RU/mL, measured using a second-generation C-terminal enzyme-linked immunosorbent assay FGF23 assay).

**Table 1.** Laboratory tests at the time of presentation for metabolic bone disease

Tests	Reference range	Results
BUN	8-24 mg/dL (SI: 2.9-8.6 mmol/L)	14 mg/dL (SI: 5.0 mmol/L)
Creatinine	0.74-1.35 mg/dL (SI: 65-119 μmol/L)	0.55 mg/dL (SI: 48.6 μmol/L) (L)
Total CO_2_	22-29 mmol/L	28 mmol/L
Calcium	8.6-10.2 mg/dL (SI: 2.15-2.55 mmol/L)	9.0 mg/dL (SI: 2.25 mmol/L)
Albumin	3.5-5.0 g/dL (SI: 35-50 g/L)	4.6 g/dL (SI: 46 g/L)
Phosphate (inorganic phosphorus)	2.5-4.5 mg/dL (SI: 0.81-1.45 mmol/L)	1.4 and 1.6 mg/dL (SI: 0.45 and 0.52 mmol/L) (L)
PTH intact	15-65 pg/mL (SI: 1.6-6.9 pmol/L)	141 pg/mL (SI: 14.95 pmol/L) (H)
ALP	40-129 U/L (SI: 0.67-2.15 μkat/L)	197 and 245 U/L (SI: 3.29 and 4.09 μkat/L) (H)
25(OH)D	30-100 ng/mL (SI: 75-250 nmol/L)	34 ng/mL (SI: 85 nmol/L)
1,25(OH)_2_D	18-72 pg/mL (SI: 43-87 pmol/L)	16 pg/mL (SI: 38.4 pmol/L) (L)
FGF23	<180 RU/mL	356 RU/mL (H)
TSH	0.3-4.2 mIU/L	1.78 mIU/L
Total testosterone	300-1000 ng/dL (SI: 10.4-34.7 nmol/L)	358 ng/dL (SI: 12.43 nmol/L)
Random urinary phosphate	No fixed adult reference range	80.4 mg/dL (SI: 2.59 mmol/L)
Random urinary creatinine	20-300 mg/dL (SI: 1.8-26.5 mmol/L)	121 mg/dL (SI: 10.69 mmol/L)
TmP/GFR	2.6-4.4 mg/dL (SI: 0.8-1.35 mmol/L)	1.24 mg/dL (SI: 0.4 mmol/L) (L)
Fractional tubular reabsorption of phosphate	85%-95%	77% (L)
24-h urinary calcium	100-300 mg/24 h (SI: 2.5-7.5 mmol/24 h)	48 mg/24 h (SI: 1.2 mmol/24 h) (L)
24-h urinary creatinine	1.4-2.6 g/24 hours (SI: 14-26 mmol/24 h)	1.56 g/24 hours (SI: 13.79 mmol/24 h)
Urinary calcium/creatinine ratio	<100 mg/g creatinine (SI: <0.25 mmol/mmol creatinine)	31 mg/g creatinine (SI: 10.95 mmol/mmol creatinine)

The pronounced low phosphate level, 1,25(OH)_2_D, TmP/GFR, and fractional tubular reabsorption of phosphate, with elevated FGF23, are compatible with tumor-induced osteomalacia.

Abbreviations: 25(OH)D, 25-hydroxyvitamin D; ALP, alkaline phosphatase; BUN, blood urea nitrogen; CO_2_, carbon dioxide; FGF23, fibroblast growth factor 23; PTH, parathyroid hormone; TmP/GFR, tubular maximum phosphate reabsorption per glomerular filtration rate; TSH, thyrotropin.

During his evaluation, a 1.9-cm right-sided thyroid nodule was detected and diagnosed as papillary thyroid carcinoma, and the patient underwent hemithyroidectomy; this led to delaying functional imaging for tumor localization.

**Figure 1.**
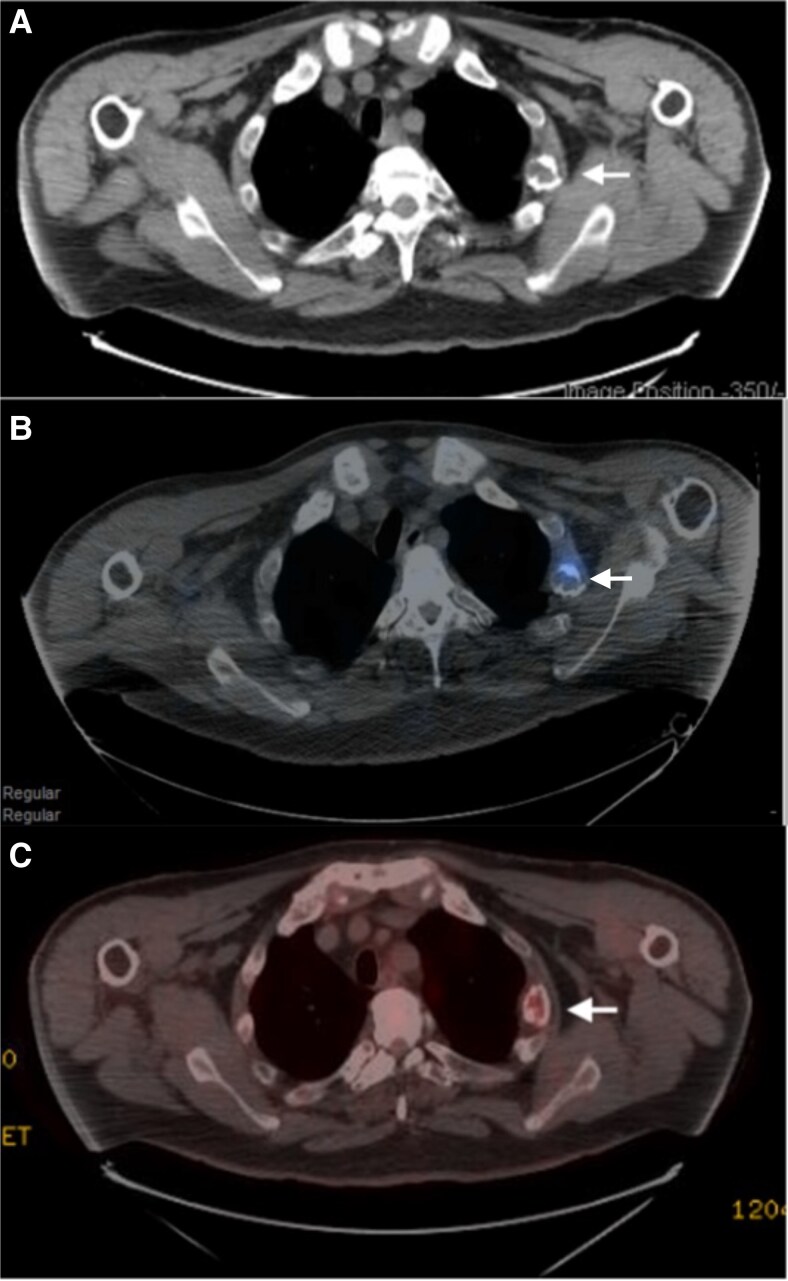
Anatomical and fused functional imaging studies. A, Computed tomography (CT) of the chest showed an expansile lytic lesion with soft tissue component associated with a healing callus on the left lateral third rib. B, Fused octreotide single photon emission (SPECT)/CT showed increased uptake in the lytic lesion on the left third rib. C, ^18^F-fluorodeoxyglucose (^18^F-FDG) positron emission tomography (PET)/CT also showed increased uptake in the same area, concordant with the octreotide scan, suspicious for a phosphaturic mesenchymal tumor.

Initial CT of the chest, abdomen, and pelvis revealed multiple healing rib fractures with nonspecific expansile lytic change in the left lateral third rib. Subsequent octreotide scan and whole-body ^18^F-fluorodeoxyglucose (^18^F-FDG) PET/CT both demonstrated low-grade radiotracer uptake located at the site of a healing fracture in the left lateral third rib, which was suspected to represent the PMT (Fig. 1A-1C).

## Treatment

The patient was initially treated with escalating doses of calcitriol and potassium phosphate; however, hypophosphatemia persisted (1.8 mg/dL [SI: 0.58 mmol/L]). Given persistent hypophosphatemia and nonspecific findings on initial anatomical imaging, with delayed functional imaging for localization, burosumab was initiated and titrated to 0.75 mg/kg every 2 weeks. This led to normalization of phosphorus level 2.5 mg/dL (SI: 0.81 mmol/L) and partial improvement in his weakness and energy.

**Figure 2.**
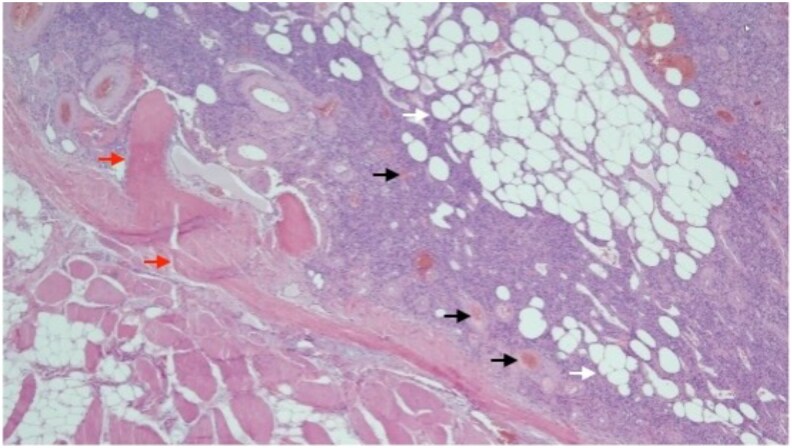
The histology of phosphaturic mesenchymal tumor. It showed proliferation of bland, spindle-shaped neoplastic cells, with prominent vasculature (black arrow) and extracellular matrix (red arrows) with adipocyte infiltration (white arrow).

After tumor localization on functional imaging, the patient underwent surgical tumor resection. Burosumab was continued, and the last dose was given 1 day before surgery. A red-brown tumor measuring 2.2 × 1.8 × 0.6 cm was resected along with adjacent bone fragments. Histopathologic examination revealed a proliferation of bland, spindle-shaped neoplastic cells with prominent vasculature and an extracellular matrix containing adipocytic infiltration, consistent with a PMT (Fig. 2). Positive FGF23 messenger RNA expression was confirmed in the neoplastic cells by chromogenic in situ hybridization.

## Outcome and Follow-up

The patient developed transient mild hypocalcemia postoperatively, with a nadir postoperative day 1 with calcium levels of 7.7 mg/dL (SI: 1.92 mmol/L) (reference range, 8.6-10.2 mg/dL [SI: 2.15-2.55 mmol/L]), while magnesium remained normal at 2.0 mg/dL (SI: 0.82 mmol/L) (reference range, 1.6-2.6 mg/dL [SI: 0.66-1.07 mmol/L]). He was started on calcium carbonate 1200 mg daily and vitamin D_3_ 2800 IU daily. Serum calcium gradually improved during hospitalization after surgery to 8.1 mg/dL (SI: 2.02 mmol/L).

**Figure 3.**
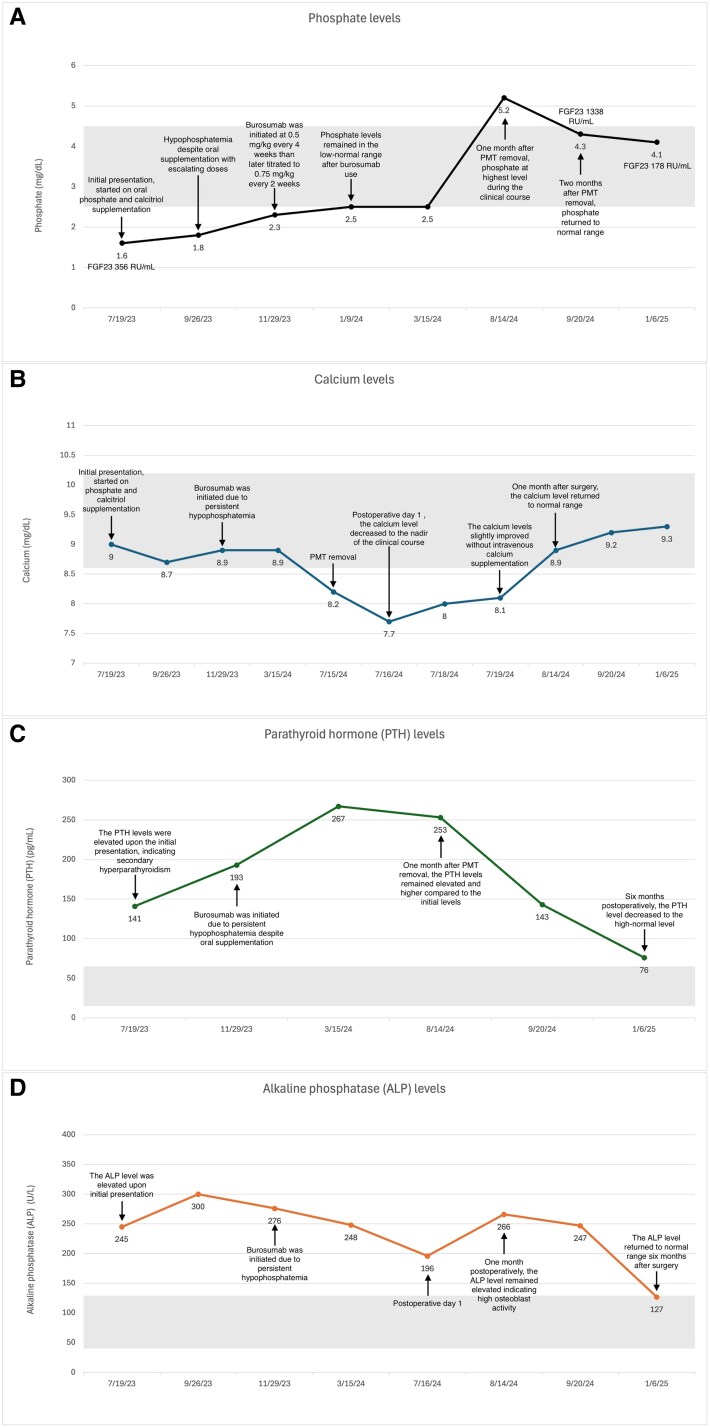
The trend of phosphate, calcium, parathyroid hormone (PTH), and alkaline phosphatase (ALP) from the initial evaluation, alongside critical clinical events. PMT, phosphaturic mesenchymal tumor; FGF23, fibroblast growth factor 23. A, The trend of phosphate levels. Serum phosphate levels remained low despite oral supplements and returned to the low-normal range after burosumab. The highest phosphate level was recorded 1 month after surgery. The gray area in the graph represents the normal reference range, 2.5 to 4.5 mg/dL (SI: 0.81-1.45 mmol/L). B, The trending of calcium levels. The calcium level decreased to nadir postoperative day 1 and slightly improved the subsequent days. The calcium level normalized at 1 month follow-up. The gray area in the graph represents the normal reference range, 8.6 to 10.2 mg/dL (SI: 2.15-2.55 mmol/L). C, The trend of PTH levels. The PTH levels were elevated at initial presentation and remained elevated after surgery. The gray area in the graph represents the normal reference range, 15 to 65 pg/mL (SI: 1.6-6.9 pmol/L). D, The trend of ALP levels. ALP elevation was observed at initial evaluation and persisted throughout the clinical course until 6 months after surgery. The gray area in the graph represents the normal reference range, 40 to 129 U/L (SI: 0.67-2.15 μkat/L).

At 1 month postoperatively, he reported improved energy and mobility. Serum calcium returned to normal range (8.9 mg/dL [SI: 2.23 mmol/L]), and phosphate was in the high-normal range (5.2 mg/dL [SI: 1.68 mmol/L]). ALP and PTH remained elevated at this time and gradually declined to near-normal levels by 6 months postoperatively (Fig. 3).

FGF23 levels increased markedly from 356 RU/mL at initial evaluation to 1338 RU/mL 2 months postoperatively, and normalized to 178 RU/mL by 6 months. C-telopeptide was elevated at 1 month and 6 months postoperatively (2891 and 705 pg/mL [SI: 2891 and 705 ng/L]) (reference range, 60-700 pg/mL [SI: 60-700 ng/L]).

BMD also improved substantially at 6 months, showing normalization at the left femoral neck (T-score −0.5) and total hip (T-score +1.0), and a marked increase in lumbar spine BMD (L1-L4 T-score +6.9).

## Discussion

Burosumab use before PMT resection is rare. It has primarily been used in patients with unresectable or recurrent tumors, where it improves phosphate homeostasis and TmP/GFR, alleviates bone pain, enhances osteoid mineralization, and promotes fracture healing, with reported reduction in pain and fatigue [8, 9]. A recent case report described 2 cases treated with burosumab before surgical resection. Both cases had classic TIO features, including hypophosphatemia, low TmP/GFR, and elevated FGF23. Burosumab was initiated due to failure to localize tumors on initial imaging and discontinued 1 to 2 months before surgery. Postoperatively, serum phosphate and TmP/GFR normalized, while FGF23 remained elevated for at least 2 months [3].

In our case, burosumab was used due to delayed functional imaging, while the last dose was given 1 day before surgery. Serum phosphate normalized postoperatively at 1 month without supplementation, and BMD improved remarkably by 6 months. FGF23 remained elevated at 2 months postoperatively, even higher compared to the preoperative level, but normalized by 6 months.

Burosumab has a known effect on FGF23 measurements, and it has been reported to cause more than 500-fold median elevation of both intact and C-terminal FGF23 in vivo following burosumab administration [10]. The mean half-life of burosumab is 16.4 days [11]. The prolonged effect of burosumab on FGF23 assays was observed in a previous report and in our case, and could persist for months even after successful tumor resection [3]. FGF23 levels should be interpreted with caution and should be expected to remain elevated for months if patients received burosumab. Serum phosphate, TmP/GFR, and BMD can be used as alternative markers to monitor treatment response.

A high bone turnover state resembling a hungry bone–like phenomenon has been described in several reports. Rendina et al [4] observed a postoperative increase in ALP with a mild decrease in ionized calcium and magnesium during the first 60 days. Kilbane et al [5] reported 2 cases with elevated PTH, ALP, and C-telopeptide levels, and normalization of FGF23 and 1,25(OH)_2_D after tumor removal; both patients had secondary hyperparathyroidism preoperatively. Kumar and Diamond [6] also described a young man with TIO who developed severe symptomatic hypocalcemia 3 weeks after surgery, requiring high-dose calcitriol and calcium supplementation. In a cohort monitoring postoperative outcomes in 117 TIO patients, serum phosphate and TmP/GFR normalized within 7 days, with peak phosphate at 1 month, and rapid decreases in FGF23 within 1 day. Persistent elevations in ALP and PTH for 1 to 3 months, along with transient mild hypocalcemia, were also reported. Before surgery, 53.8% (63/117) of the patients had elevated PTH, and 93.2% (109/117) had elevated ALP [12].

The pathophysiology of this high turnover state is thought to reflect rapid osteoid mineralization due to restored phosphate availability and decreased FGF23 levels, in contrast to the reduced bone resorption observed in hungry bone syndrome following parathyroidectomy [4-6].

Our case exhibited biochemical features similar to previously reported TIO cases, including secondary hyperparathyroidism and elevated ALP at presentation. In a previous cohort study, secondary and tertiary hyperparathyroidism were reported in 41.6% and 3.5% of TIO cases, respectively [13]. These subgroups were associated with longer disease duration compared with patients without hyperparathyroidism, as well as higher rates of oral phosphate and calcitriol supplementation and elevated 1,25(OH)_2_D level [13].

After tumor removal, transient hypocalcemia developed postoperative day 1 and resolved with calcium and vitamin D supplementation within 1 month. PTH and ALP levels both remained elevated for several months, consistent with a high bone turnover state, and normalized by 6 months postoperatively. Substantial improvement in BMD was observed after tumor resection, particularly in the lumbar spine (T-score improved from −2.6 to +6.9). This improvement in BMD and normalization of phosphate levels reflected recovery from TIO, despite persistent elevated FGF23. These findings are consistent with prior case series, which reported marked BMD gains, most prominently in the lumbar spine, from a *Z*-score of −2.80 ± 1.60 at baseline to +1.75 ± 1.42 after tumor removal [14].

## Learning Points

- Burosumab can interfere with both intact and C-terminal FGF23 assays in vivo, and this effect may persist for several months. It is not routinely recommended if the culprit tumor can be localized and resected. In patients who have received burosumab preoperatively, alternative markers, including serum phosphate, TmP/GFR, and BMD, should be used to monitor disease resolution. Clinicians should interpret FGF23 levels cautiously if anti-FGF23 therapy is administered near the time of surgery.
- A high bone turnover state may occur after tumor removal, characterized by persistent elevations in ALP and hyperparathyroidism with transient hypocalcemia, resembling hungry bone syndrome after parathyroidectomy. The mechanism is thought to result from rapid mineralization following restored phosphate availability and decreased FGF23 levels. Awareness of these postoperative biochemical changes is essential for understanding the expected course of recovery in TIO.

## Data Availability

Original data generated and analyzed for this case report are included in this published article.

## Abbreviations

^18^F-FDG: ^18^F-fluorodeoxyglucose
1,25(OH)_2_D: 1,25-dihydroxyvitamin D
25(OH)D: 25-hydroxyvitamin D
ALP: alkaline phosphatase
BMD: bone mineral density
FGF23: fibroblast growth factor 23
PET/CT: positron emission tomography/computed tomography
PMTs: phosphaturic mesenchymal tumors
PTH: parathyroid hormone
TIO: tumor-induced osteomalacia
TmP/GFR: tubular maximum phosphate reabsorption per glomerular filtration rate
